# A retrospective survey of dengue fever among Japanese individuals staying in Manila, Philippines

**DOI:** 10.1186/s41182-016-0027-4

**Published:** 2016-08-17

**Authors:** Atsuo Hamada, Yuki Tada, Shinji Fukushima, Hidemi Murata, Hirohisa Kikuchi

**Affiliations:** 1 Traveller’s Medical Center, Tokyo Medical University Hospital, 6-7-1 Nishi-shinjyuku, Shinjyuku-ku, Tokyo, Japan 160-0023; 2 Japan Overseas Medical Fund, 2-4-2 Nishi-shinbashi, Minato-ku, Tokyo, Japan 105-0003; 3 Japan Overseas Medical Fund stationed in medical clinic of The Japanese Association Manila INC, 23F Trident Tower, 312 Sen. Gill Puyat Ave. Salcedo village, Makati City, Philippines

**Keywords:** Dengue fever, Japanese residents, Manila, Prevention

## Abstract

Dengue fever is a serious concern for Japanese people staying in Southeast Asia. In order to implement necessary prophylactic measures for dengue fever in this population, we investigated the characteristics of dengue fever among Japanese nationals living in Manila, Philippines. From 2012 to 2015, 175 Japanese expatriates were diagnosed with dengue fever at the medical clinic of the Japanese Association Manila, Inc. Most of the patients were employees of Japanese companies and their families and were long-term residents of Manila. Most patients were either <10 years or in their 30s to 40s. Two patients (1.1 %) were diagnosed with dengue hemorrhagic fever. No deaths due to dengue fever were reported. The reported number of patients with dengue fever has shown a decreasing trend: from 55 cases in 2012 to 53 in 2013, 31 in 2014, and 36 in 2015. The results of this survey could be useful for the development of effective dengue fever preventive measures such as health education and provision of information among not only Japanese but also other foreigners residing in endemic areas.

## Background

Dengue fever is of concern for Japanese people residing in areas of Southeast Asia. Prevention of bites from infected vector mosquitoes is strongly recommended to prevent transmission of dengue fever in endemic areas.

From 2010 onward, the reported number of imported dengue cases in Japan increased to more than 200 cases per year, primarily due to outbreaks in Southeast Asian countries [1–3]. Furthermore, in 2014, 162 autochthonous dengue fever cases, probably originating from imported cases, were reported in Tokyo and other areas in Japan [3, 4].

Japanese law requires that all domestically diagnosed dengue fever cases be reported to the Ministry of Health, Labour and Welfare. However, the epidemiological status of dengue fever in Japanese people residing in endemic regions remains unknown. In order to implement appropriate prophylactic measures for dengue fever, such as health education and provision of information, we investigated the status of dengue fever among Japanese expatriates living in Manila, Philippines.

## Methods

Japanese individuals living in the Philippines who visited the medical clinic of the Japanese Association Manila, Inc., run by the Japan Overseas Medical Fund, were recruited to participate in this survey. This clinic is located in the business area of Manila (Makati city), where many Japanese companies are located. Approximately 10,000 Japanese expatriates live in the Manila metropolitan area (information from the Japanese Embassy in the Philippines), and the majority of them visit this clinic for their primary health care. This clinic sees 9000–10,000 Japanese patients each year. The clinic summarizes information regarding infectious diseases among Japanese patients visiting this clinic each month, which is reported to the Tokyo headquarters of the Japan Overseas Medical Fund. These aggregated data summarize the epidemiological status of dengue fever (patient age, sex, and reason for staying in Manila) as well as the seasonal onset of the disease. The reports describe only the numbers of patients and disease conditions without including individually identifiable patient information. We accessed these aggregate reports with permission from the Japan Overseas Medical Fund and performed a retrospective survey by analyzing data from Japanese patients diagnosed with dengue fever between January 2012 and December 2015.

Patients were tested for dengue fever using commercial dengue nonstructural protein 1 (NS1) antigen (SD Bioline Dengue NS1 Ag: Standard Diagnostics, Inc.), dengue-specific IgM antibody (SD Bioline Dengue IgG/IgM: Standard Diagnostics, Inc.), and dengue-specific IgA antibody (Assure Dengue IgA Rapid Test: MP Biomedicals) assays [5]. Patients were diagnosed with dengue fever based on positive results in any one of examinations. More specifically, NS1 antigen analysis is considered the first-line test for clinical diagnosis of dengue fever. Patients with negative NS1 findings were further subjected to dengue-specific IgM or IgA antibody assays; they were considered positive for infection based on positive results in either of these tests. Dengue hemorrhagic fever was diagnosed according to standards established by the Ministry of Health, Labour and Welfare in Japan [2].

This study was performed following review and approval from the Ethics Board of Tokyo Medical University (No. 3080).

## Results

During the 4-year study period (January 2012 to December 2015), 175 Japanese patients were diagnosed with dengue fever at the medical clinic of the Japanese Association Manila, Inc. (Table 1). For making diagnosis in these patients, NS1 antigen, IgM antibody, and IgA antibody tests were performed for 127, 24, and 24 patients, respectively. Of these patients, 105 were male and 70 were female. Their ages ranged from 1 to 80 years, but most patients were aged <10 years or in their 30s and 40s. The major reason for staying in Manila was prolonged dispatch by companies (88 cases) and their families (81 cases).

**Table 1:** Number of Japanese dengue patients visiting the Japanese Association Manila, Inc., medical clinic (2012–2015)

	Total	Year			
		2012	2013	2014	2015
Number of patients	175	55	53	31	36
Sex					
Male	105 (60.0 %)	32	36	21	16
Female	70	23	17	10	20
Age (years)					
<10	23 (13.1 %)	11	7	3	2
10~19	12 (6.9 %)	5	2	2	3
20~29	12 (6.9 %)	6	2	3	1
30~39	52 (29.7 %)	14	17	11	10
40~49	42 (24.0 %)	13	13	7	9
50~59	22 (12.6 %)	1	8	5	8
≥60	12 (6.9 %)	5	4	0	3
Purpose for visit					
Sent by the company	88 (50.3 %)	25	32	17	14
Family members of company employee	81 (46.3 %)	29	20	13	19
Other	6 (3.4 %)	1	1	1	3
Severity					
Hemorrhagic fever	2 (1.1 %)	2	0	0	0
Hospitalized	49 (28.0 %)	16	18	10	5
Mortality	0	0	0	0	0

Two patients (1.1 %), a woman in her 30s and a teenage girl, were diagnosed with dengue hemorrhagic fever but recovered after hospitalization. No deaths due to dengue fever were reported. Among all patients, 49 (28.0 %) were hospitalized, most of whom were children <10 years of age (9 cases) or adults in their 30s (21 cases).

Almost equal numbers of dengue fever cases were reported in 2012 (55 cases) and 2013 (53 cases), but the numbers decreased somewhat in 2014 (31 cases) and 2015 (36 cases) (Table 1). The percentages of patients with dengue fever among all patients who visited the clinic have also decreased since 2014, dropping from 0.56 % in 2012 to 0.50 % in 2013, 0.27 % in 2014, and 0.29 % in 2015.

**Fig. 1:**
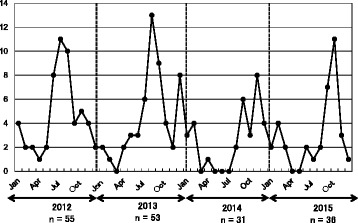
Monthly number of Japanese dengue patients visiting the Japanese Association Manila, Inc., medical clinic

Many of the cases in 2012 and 2013 were reported during the rainy season (between June and August) (Fig. 1). However, in 2014 and 2015, fewer cases were reported in the rainy season in contrast to the increased number of patients reported between October and November.

## Discussion

A survey of European international travelers indicated that 1–3 % of the travelers contracted dengue virus infections during a 1-month stay in tropical areas [6]. In Japan, more than 200 patients with imported dengue fever have been reported annually since 2010 [3]. These results suggest that international travelers visiting tropical areas are at high risk for dengue fever; however, to our knowledge, no investigations have reported the rates of dengue fever among Japanese expatriates living in endemic areas. In the present report, we investigated the status of dengue fever among Japanese people living in Manila, Philippines.

In the current study, a total of 175 Japanese people were diagnosed with dengue fever at the medical clinic of the Japanese Association Manila, Inc., between 2012 and 2015. Most of the dengue patients were company employees sent from Japan, as well as their families. Sixty percent of the patients were male, and many were <10 years or in their 30s and 40s. A survey of imported dengue fever cases in Japan (*n* = 762, 2011–2014) showed that about 60 % of patients were male, similar to the percentage observed in the present survey; however, a large proportion of the patients were in their 20s, in contrast to the results of the present survey [3]. In the present investigation, two (1.1 %) of the 175 cases were diagnosed with dengue hemorrhagic fever, a lower incidence rate than the 5 % reported in the abovementioned study on imported dengue cases.

There have been several surveys on dengue fever among expatriates. In an Israeli survey of long-term travelers (mean duration of stay, 5.3 months), the ratio of dengue fever seroconversion was 6.7 % [7]. A New Zealand survey of long-term development aid workers in Southeast Asia (mean duration of stay, 22 months) reported a dengue fever seroconversion rate of 15.1 % [8]. Although we were unable to obtain the incidence rate in our present study because the size of the population was unknown, the results of this study and others indicate that dengue fever is an infectious disease that affects expatriates in endemic areas at a high frequency.

In the present study, the annual numbers of dengue cases decreased from more than 50 in 2012 and 2013 to 30 to 40 in 2014 and 2015. The reported incidence of dengue fever has decreased considerably in the Philippines since 2010, to just over 100,000 patients diagnosed annually [9, 10]. However, the Regional Office for the Western Pacific of the World Health Organization summarized the recent trend of dengue incidence in the Philippines [11], reporting a rapid increase in the numbers of patients in 2012 and 2013, to approximately 180,000 and 170,000 cases, respectively. These numbers decreased to approximately 110,000 in 2014 but increased again to 160,000 in 2015 [11]. In the present survey, the dengue incidence rate among Japanese people in Manila was lower in 2014, probably because of the ameliorated epidemiological status nationwide. Also, since 2012, we have offered periodic educational lectures focused on dengue prevention for Japanese people living in Manila. These awareness activities might have contributed to the implementation of proactive preventive measures among Japanese people, resulting in a consistently low incidence in 2015.

This retrospective study was based on data available from monthly reports provided to the Japan Overseas Medical Fund by the medical clinic of the Japanese Association Manila, Inc. Therefore, we were able to access only limited patient data such as age, sex, and reasons for staying in Manila. In order to obtain more information about the epidemiological status of dengue fever among Japanese people in Manila, future prospective surveys are required. To reveal the relevant factors associated with the decreased prevalence of dengue patients since 2014, a questionnaire survey may be effective to assess changes in preventive measures against dengue fever among Japanese people.

## Conclusions

The present study reports the status of dengue fever among Japanese expatriates in Manila, Philippines. The findings of this study may be useful for the development of more effective preventive measures such as health education and provision of information for dengue fever among not only Japanese people but also other foreigners living in endemic areas.
